# Esophageal Lung in a Preterm Boy—Report on a Multidisciplinary Treatment

**DOI:** 10.1055/s-0039-3400475

**Published:** 2019-11-28

**Authors:** Enrique Villamizar, Maria Daniela Moreno Villamizar, Mauricio Pedraza Ciro, Jean Pulido, Maria Rodriguez, Juan Carlos Villamizar

**Affiliations:** 1 Department of Pediatric Surgery, Hospital Universitario Erasmo Meoz, Bogota, Colombia; 2 Department of Surgery, Universidad El Bosque Facultad de Medicina, Bogota, Colombia; 3 Registered Nurse, Morton College, Cicero, Illinois, United States; 4 Department of Medicine, Industrial University of Santander, Bogota, Colombia

**Keywords:** esophageal lung, communicating foregut malformations, bronchopulmonary malformations, recurrent pneumonia

## Abstract

Esophageal lung is a rare entity that results from embryological alterations during the formation of the ventral wall of the anterior intestine. The clinical manifestations of this pathology are vague, including respiratory or digestive symptoms, repetitive respiratory infections, dysphagia, or inability to swallow. The management is based on the exact anatomical and vascular abnormalities. We report the diagnostic and therapeutic approach in a preterm boy with esophageal lung. Also, we present a three-dimensional model for the classification of this pathology. In conclusion, the management relies on proper definition of the anatomy and the surgical strategy.

## Introduction

Esophageal lung is a rare bronchopulmonary foregut congenital malformation (BPFM). It is characterized by a communication between a lung segment, lung tissue, and/or the main bronchus with the esophagus. 1 The clinical findings vary according to the severity of the malformation; the patients may present with a chronic cough, feeding difficulty, repeated pulmonary infections, and nonspecific dyspnea, as described by Sugandhi et al and Katz et al, 2 3 some patients might even develop severe respiratory distress leading to death. In most cases worldwide, the diagnosis is suspected by the presence of hemoptysis, dysphagia, feeding-related respiratory distress, and recurrent respiratory infections. 4

Currently, there are no guidelines established for treatment. The management is determined by the aberrant anatomy presentation, knowledge, and expertise of the attending physicians. Based on literature findings, the surgical objective is aimed at separating the existing communication between the esophagus and the bronchial structure in question.

We report a case of a preterm newborn male with a diagnosis of esophageal lung type III by Srikanth et al classification, and describe the initial experience in the management of this pathology with thoracotomy.

## Case Report

**Fig. 1:**
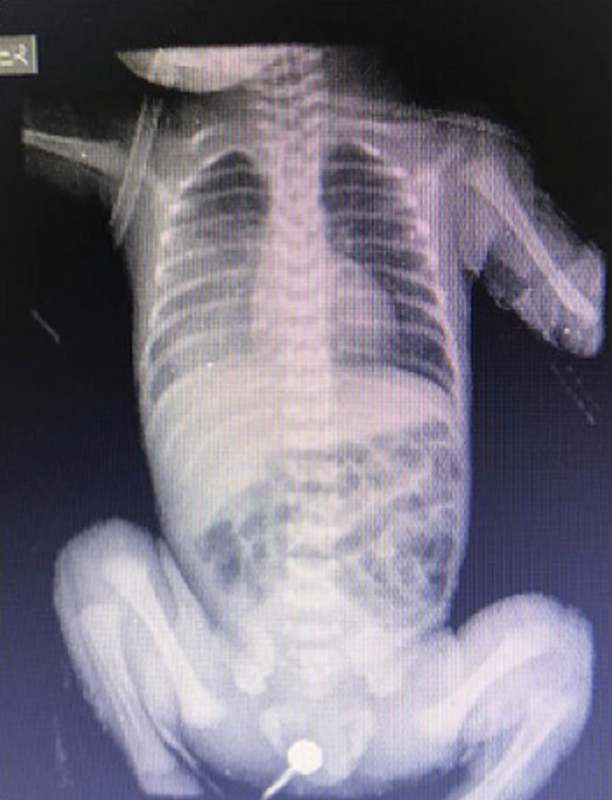
Full body X-rays showing opacities in the right hemithorax and air bronchogram.

The child was a preterm born at 31 weeks of gestational age, via emergency cesarean section due to polyhydramnios. He was a twin product of a dichorionic diamniotic pregnancy, APGAR scores were 8 at 1 minute and 10 at 5 minutes, birth weight was 1,635 g, and height 46 cm. Referred from Venezuela to our institution in Cúcuta, Colombia, the child was admitted to the pediatric intensive care unit because of high risk for septicemia and the presence of respiratory distress. The patient was intubated, and placed on mechanical ventilation due to his clinical condition. Several blood test and imaging studies were performed during hospitalization: chest X-ray showed an opacity with air bronchogram, as well as a markedly hyperinsufflated lung in the lower two-thirds of the right lung ( Fig. 1 ). Antibiotic treatment was initiated due to clinical suspicion of in utero pneumonia.

**Fig. 2:**
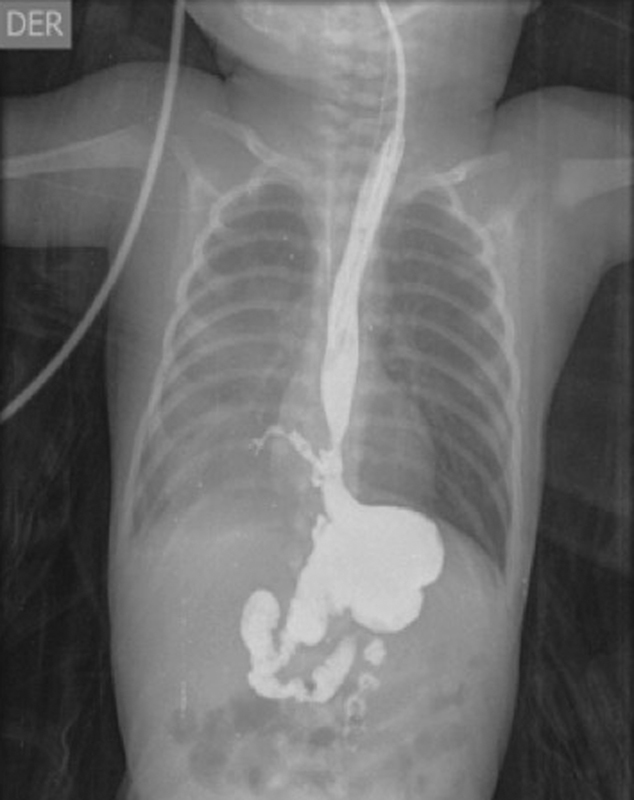
Upper digestive tract X-rays: Partial obstruction of the distal esophagus with right bronchial tree image stemming from the site of the obstruction.

Upon arrival to our institution, a multidisciplinary group, that included members of pediatric surgery, neonatology, cardiology, nutrition(ist), social work, and psychology departments, was created for the assessment and management of the patient and support to his family. He was in respiratory distress requiring supplemental oxygen and nutritionally compromised in the need of nutritional support. During hospitalization, the patient developed oral intake intolerance, the multidisciplinary group recommended an upper gastrointestinal (GI) X-ray to rule out possibility of a tracheoesophageal fistula (TEF) ( Fig. 2 ). A partial obstruction of the distal esophagus with contrast filling of the right bronchial tree and connection to the site of the obstruction was observed.

**Fig. 3:**
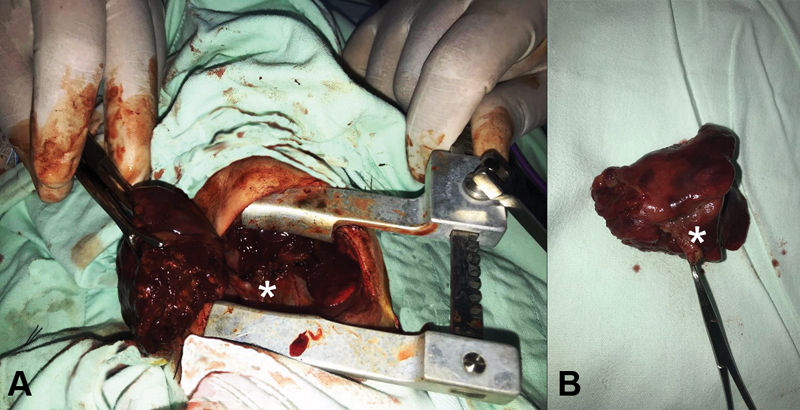
Surgical findings of bronchopulmonary esophageal fistula indicated with white asterix.

On the ninth day of life in the multidisciplinary assessment and suspicion of BPFM, it was decided to perform a computed tomographic (CT) scan which showed a solidification of right basal lung tissue. An echocardiogram showed no vascular anomalies. Because of these findings, a right thoracotomy and right inferior lobectomy were performed, resecting an anomalous pulmonary tissue from its stemming point on the esophagus as well as an esophageal mass ( Fig. 3 ), resulting in resolution of the obstructive esophageal stenosis, and regaining of the oral intake tolerance.

The histopathology showed a hypoplastic lung with bronchus lined by respiratory epithelium and a bronchogenic cyst, lymphocytic infiltrate, presence of lymphoid follicles, and well-formed germinal centers.

VACTERL sequence was ruled out. Postoperative course on day 1 was uneventful, and enteral nutrition via nasogastric tube was initiated on postoperative day 2. On postoperative day 5, an upper GI contrast study confirmed no leak from the repaired esophagus; the mid-esophageal dilatation had also subsided significantly with normal dye passage distally into the stomach, then oral intake was initiated. The patient had difficulty tolerating oral intake, and required to be hospitalized for 1 month to improve his nutritional status. The child was then discharged on oral feeds. The patient presented recurrent pulmonary infection after the surgery up to the age of 10 months. The patient was discharged after adequate postoperative care, and was asymptomatic during 1-year follow-up.

## Discussion

Esophageal lung is an extremely rare type of BPFM, the incidence is described mainly in sporadic case reports throughout the world, without any predominant geographical area.

This anomaly is described by some as being a part of the spectrum of the more severe malformation of tracheal agenesis, with the distal trachea, the bronchi, or both arising separately from the esophagus. The first case was described by Klebs in 1874, 4 5 then in 1968, Gerle et al raised the use of the terminology of communicating malformations of the anterior bowel. 6 However, it was not until 1996 when Floyd et al made the last characterization as part of tracheal and esophageal malformations. 5 Possible etiological causes on the formation of the communicating epithelium from the anterior intestine between the squamous and the respiratory epithelium, even though a clear, established etiology, has not been described. 7 8

**Fig. 4:**
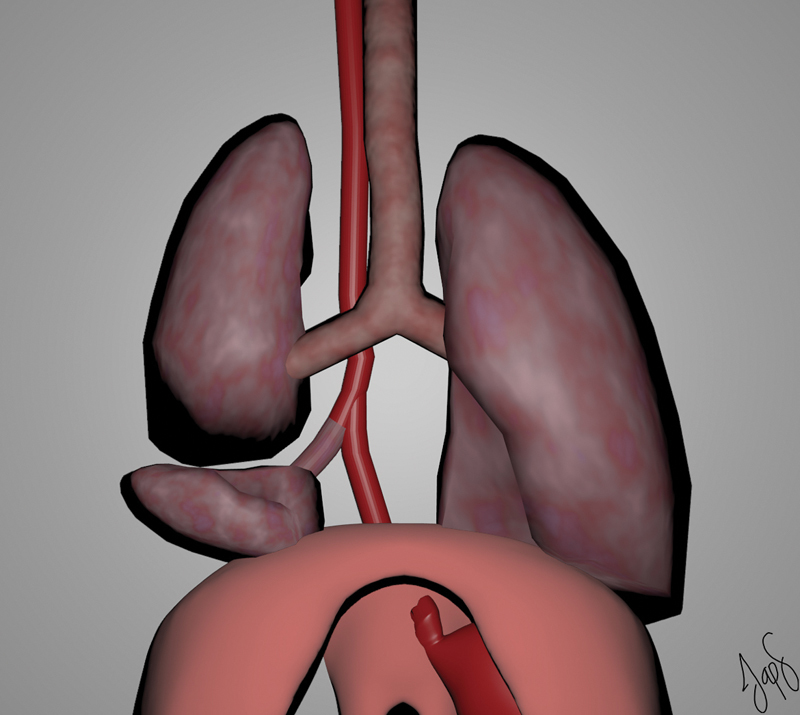
Three-dimensional (3D) model of esophageal lung type III by Srikanth et al classification.

The respiratory primordium grows caudally while the esophageal primordium grows cephalically. At the crossing of these structures during growth, it is believed that the formation of this communication is formed by small early structures of the previously mentioned tissues. Srikanth et al show that the esophageal lung entity is predominant on the right lung in almost 94% of cases. 6 9 Our patient presented an esophageal lung type III as Srikanth et al described ( Fig. 4 ).

Patients with this condition are usually diagnosed during the neonatal period who develop lower respiratory tract infections, or where a genetic anomaly is suspected as were presented in our patient. The main features of esophageal lung are the complete involvement of the affected lung and its circulation is provided mainly by the pulmonary artery. These features allow the differentiation from pulmonary sequestration, according to the description by Sugandhi et al. 2

X-ray, barium contrast studies, CT scan angiography, and magnetic resonance imaging (MRI) angiography could be used in the diagnostic approach, and could also provide an approximation on differential diagnoses, such as bronchopulmonary sequestration and enteric duplication cysts. 10 11 Colleran et al 12 conclude that lung opacification, ipsilateral mediastinal shift, an abnormal carina, and anomalous vascular anatomy suggest an esophageal bronchus or an esophageal lung on CT, findings compatible with what was presented in the case. Nevertheless, MRI and fibrobronchoscopy should be used as a complementary study that shows the malformation added to vascular studies and the respiratory tree anatomy. 13 Early diagnosis of esophageal bronchus might prevent complications, such as aspiration and infection, which can allow for parenchymal sparing surgery as opposed to pneumonectomy. 12

Multiple malformations in association with this pathology, among them, esophageal atresia, stenosis or duodenal atresia, congenital heart disease, costovertebral malformations, and “pulmonary artery sling,” 14 15 are present and so the VACTERL (vertebral defects, anal atresia, cardiac defects, TEF, renal anomalies, and limb abnormalities) association should be considered, as it was ruled out in our patient. 4 11 12 16

Initially, resection has been described for management of abnormal communicating tissue in addition to removal of the hypoplastic lung that has been destroyed by recurrent infections; with repairing of the esophageal communication. 16 17

Lallemand et al reported on successful bronchus implantation in neonatal patients, which has shown a good clinical course and postoperative function, mainly in patients diagnosed early in the neonatal period. 1 11 Traditional management consists of surgical excision of this abnormal communication. 2 Molina et al showed that management through thoracoscopy in a case report from their experience has been followed and is feasible when there are no vascular anomalies. However, in the reviewed literature worldwide there are few such cases. 8

Compared with conventional open repair, the main advantages of thoracoscopy are the superior visualization and avoidance of the thoracotomy incision. However, the hemodynamic and respiratory status of our patients do not allow a minimally invasive approach, so it must be taken into account when deciding the type of approach. 18 19 20

In conclusion, the management of esophageal lung is challenging. We believe that, if feasible, however, all clinical and surgical variables should be taken into account. Different types of management have been described as tracheobronchial anastomosis with the preservation of the lung to allow the best quality of life to the affected child as were reported by Ichino et al. 21 However, if this approach is not possible for anatomical reasons, a reasonable treatment plan is an early closure and division of the esophageal bronchus, followed by delayed pneumonectomy of the esophageal lung. Ichino et al explain that this type of management allows a slow adaptation of thoracic anatomy to reduce the risk of postpneumonectomy syndrome. 21

## Conclusion

Esophageal lung is a rare entity, part of the spectrum of BPFM, presented in the neonatal period. The clinical presentation of this entity is a respiratory distress secondary to recurrent respiratory infections and/or swallowing difficulty. In the majority of the cases, the management is directed to the excision of the abnormal lung, nevertheless proper anatomy identification is required to develop an adequate plan for a surgical approach. This type of pathology requires a multidisciplinary approach and a careful follow-up.

## Notes

New Insights and the Importance for the Pediatric Surgeon Esophageal lung is uncommon in children and is extremely rare with few cases reported in literature. The management of esophageal lung is a challenging subject. It represents a diagnostic challenge and should be included in the differential diagnosis of nonspecific respiratory symptoms in newborn.
